# Use of a Sustainable Livelihood Framework–Based Measure to Estimate Socioeconomic Impact of Tuberculosis on Households

**DOI:** 10.1093/cid/ciad273

**Published:** 2023-05-03

**Authors:** Collins Timire, Debora Pedrazzoli, Delia Boccia, Rein M G J Houben, Rashida A Ferrand, Virginia Bond, Katharina Kranzer

**Affiliations:** Department of Clinical Research, Faculty of Infectious and Tropical Diseases, London School of Hygiene and Tropical Medicine, London, United Kingdom; AIDS and TB Department, Ministry of Health and Child Care, Harare, Zimbabwe; The Health Research Unit, Biomedical Research and Training Institute, Harare, Zimbabwe; Department of Infectious Disease Epidemiology, London School of Hygiene and Tropical Medicine, London, United Kingdom; Department of Infectious Disease Epidemiology, London School of Hygiene and Tropical Medicine, London, United Kingdom; Department of Infectious Disease Epidemiology, London School of Hygiene and Tropical Medicine, London, United Kingdom; Department of Clinical Research, Faculty of Infectious and Tropical Diseases, London School of Hygiene and Tropical Medicine, London, United Kingdom; The Health Research Unit, Biomedical Research and Training Institute, Harare, Zimbabwe; Social Science Unit, Zambart, Lusaka, Zambia; Department of Global Health and Development, Faculty of Public Health and Policy, London School of Hygiene and Tropical Medicine, London, United Kingdom; Department of Clinical Research, Faculty of Infectious and Tropical Diseases, London School of Hygiene and Tropical Medicine, London, United Kingdom; The Health Research Unit, Biomedical Research and Training Institute, Harare, Zimbabwe; Division of Infectious Diseases and Tropical Medicine, Medical Center of the University of Munich, Munich, Germany

**Keywords:** well-being, tuberculosis, sustainable livelihood, coping strategies, impoverishment

## Abstract

Tuberculosis (TB) disproportionally affects impoverished members of society. The adverse socioeconomic impact of TB on households is mostly measured using money-centric approaches, which have been criticized as one-dimensional and risk either overestimating or underestimating the true socioeconomic impacts of TB. We propose the use of the sustainable livelihood framework, which includes 5 household capital assets (human, financial, physical, natural, and social) and conceptualizes that households employ accumulative strategies in times of plenty and coping (survival) strategies in response to shocks such as TB.

The proposed measure ascertains to what extent the 5 capital assets are available to households affected by TB as well as the coping costs (reversible and nonreversible) that are incurred by households at different time points (intensive, continuation, and post–TB treatment phase). We assert that our approach is holistic and multidimensional and draws attention to multisectoral responses to mitigate the socioeconomic impact of TB on households.

## Teaser

Tuberculosis leads to income and nonincome losses for people and their households. Current measures are one-dimensional and money centric and are benchmarked against income. We propose a multidimensional measure that captures both income and nonincome losses due to TB.

## Graphical Abstract

**Graphical Abstract:**
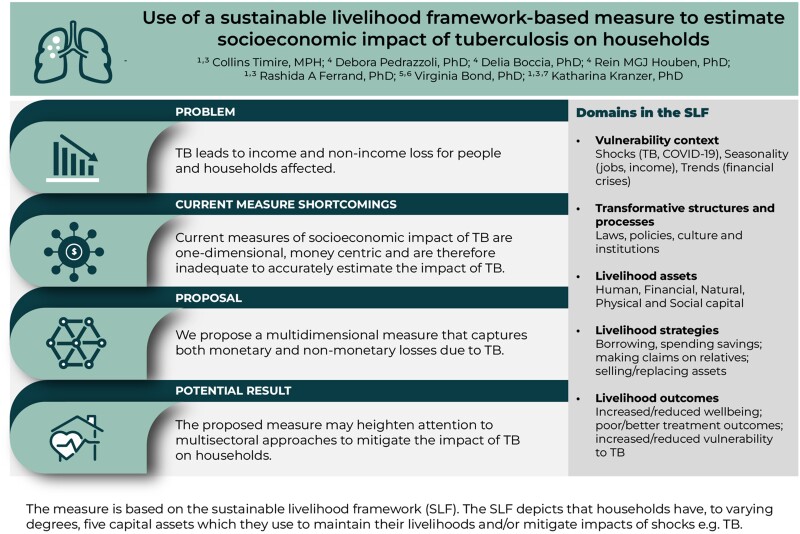

Tuberculosis (TB) affects the poorest in society, often leading to substantial socioeconomic consequences [1]. These include monetary losses due to sickness, time spent seeking healthcare, and loss of productive time by household members caring for sick people, as well as nonmonetary losses (eg, stigma, and worsening social relations) [2]. Potter and White argued that “TB patients want to live, not merely survive” [3]. Hence, measures of socioeconomic impact of TB must capture all facets of physical, social, and financial well-being. Current quantitative measures of socioeconomic impact of TB on households are money centric and one-dimensional and exclude nonmonetary losses (eg withdrawal of children from schools) [2]. Consequently, they underestimate the impact of TB in people with poor social networks who may experience worsening social relations, including abandonment by spouses and/or family. These people may be forced to adopt coping strategies (selling assets and taking loans at exorbitant interest rates). All of these have huge impacts on livelihoods, yet they are not captured by money-centric measures. By contrast, money-centric measures tend to overestimate the impact of TB in poor households, which have little or unreliable income [4], and among people who have strong social networks. The latter draw on social networks for cash or interest-free loans to cover transport and food. Family and community members often assist by supplying food, caring for children, or attending to household chores. Hence, though households may incur high TB-related costs, the costs may be borne or mitigated by their social networks.

We propose a multidimensional measure of socioeconomic impact of TB. The measure captures monetary and nonmonetary losses and is informed by the sustainable livelihood framework (SLF) [2]. We first discuss current quantitative measures, then describe the SLF and how it can be applied to develop indicators for our measure. Next, we outline data collection and analysis, aiming for a stable and holistic measure indicative of either resilience or loss of livelihood. We conclude by summarizing how the measure can be applied to evaluate effectiveness of social protection interventions.

## CURRENT QUANTITATIVE MEASURES OF FINANCIAL PROTECTION

Measures of financial protection are money centric [5]. They include high (or catastrophic) health spending and impoverishment. The first quantifies the proportion of the population whose resources are catastrophically reduced by spending on healthcare. The latter estimates the proportion of the population that is pushed below a defined poverty line due to seeking and receiving care. The End TB Strategy indicator of TB-related catastrophic costs is measured using standardized nationally representative surveys of costs incurred by individuals and households affected by TB (in shorthand, “TB patient cost surveys”) [6]. When total (direct and indirect) costs exceed 20% of a household's annual income prior to TB, they are considered catastrophic [7]. These measures have 3 main shortcomings.

First, they are benchmarked against income, an unstable metric particularly among people who are informally employed or rely on seasonal jobs—arguably many people affected by TB. Given that poor people have little or unstable incomes, measures that are benchmarked against income tend to overestimate the impact of TB. Furthermore, benchmarking against income affects reproducibility and reliability of resultant estimates, which vary depending on how income is estimated [8, 9].

Second, impoverishment is dependent on the chosen poverty line. Poverty is multidimensional and may not correlate with poverty lines, which are one-dimensional [10].

Third, current measures are not holistic. They do not account for coping strategies and social consequences in their estimates. Poor people usually make use of all resources available to them and may spend their savings and sell assets to cope with vulnerabilities [4, 11]. Qualitative studies have described the far-reaching effects of TB on households over and above costs and income, including stigma, reduced productivity (after sale of productive assets, physical morbidity), and reduced prospects of marriage [12–14]. While opportunity or indirect costs associated with lost productivity due to illness, time spent seeking healthcare, or caring for sick people instead of working [15] can be estimated using quantitative measures described above, social impacts of TB (eg, loss of future potential earnings associated with withdrawing children from school and abandonment by spouses/family) are difficult to quantify [16]. Coping strategies are considered a better indicator of socioeconomic impact of TB compared to monetary costs [8]. Recall bias is unlikely with coping strategies since people tend to remember painful experiences (eg, distress sale of assets). Though data on coping costs are collected in TB patient cost surveys, they are not included in overall estimates of catastrophic costs.

Deeper understanding of the socioeconomic impact of TB on household well-being is likely enhanced when analyses are broadened in order to understand causes and processes of impoverishment and how households respond to shocks [17]. Measures that capture all facets of well-being, including coping costs, may provide better estimates of impact of TB. Given the centrality of nonmonetary costs (eg, coping costs), we argue that there is scope for a holistic and multidimensional measure. An example of a multidimensional measure is the validated Multidimensional Poverty Index (MPI), which captures data on health, living standards (assets), and education [18]. It quantifies household poverty more holistically than measures such as “impoverishment.” However, impact of TB is usually so sudden that the MPI may not be the ideal measure to adequately capture such an impact. For example, while the MPI ascertains school enrollment status and years of schooling for each child, these indicators may remain stable despite decreasing quality of schooling and educational attainment among children in TB-affected households. Children may transfer to cheaper schools or stop attending school temporarily to take care of siblings or sick parents. Moreover, health indicators—for example, child mortality (measured 5 years prior to a survey) and undernutrition—may not be affected by sudden shocks (eg, TB). Importantly, the MPI does not capture productive capacity of household members, changes in household income, and social networks/relations, which are often affected by TB-related stigma. Though the MPI has been used to measure poverty as a predictor for TB [19], it lacks the nuances to estimate impacts of TB. Hence, there remains a gap regarding a multidimensional measure of impact of TB on households. We believe the SLF informs such a measure [20, 21].

## THE SUSTAINABLE LIVELIHOOD FRAMEWORK

**Figure 1.**
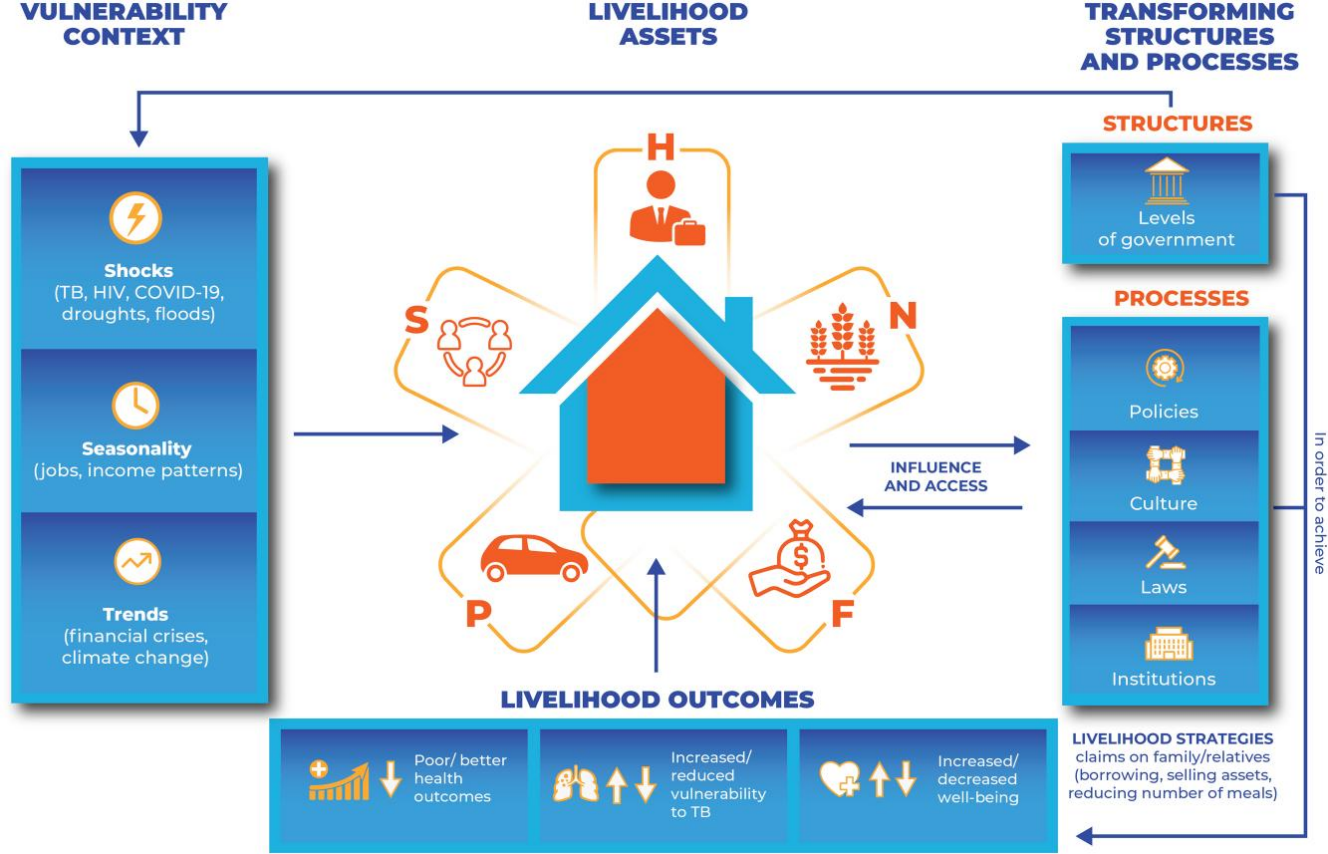
The sustainable livelihood framework. Adapted from the Department for International Development (1999). Abbreviations: COVID-19, Coronavirus Disease 2019; F, financial capital; H, human capital; HIV, human immunodeficiency virus; N, natural capital; P, physical capital; S, social capital; TB, tuberculosis.

Sustainable livelihood is when households experience shocks, recover, and retain their capacity to cope with future shocks [22]. It has been used in agroforestry to measure resilience of communities to drought and floods [23]. The SLF (Figure 1) depicts that households possess, to varying degrees, an interplay of 5 capital assets (human, financial, physical, social, and natural capital) and various livelihood strategies and activities they engage in to maintain their well-being and to cope with shocks [2] (Box 1).

Box 1: Operational Definitions

*Livelihood* refers to the skills and strategies to maintain a good life (well-being) [21].

*Claims* are demands or appeals that are made for moral and practical support, especially during times of stress or shocks. They are based on right, precedent, social convention, and moral obligation and power [23].

*Access* is the opportunity to use a resource, store, or service (transport, education, health, and markets) or to obtain information, technology, material, employment, food, or income.

*Stresses* are pressures that are continuous, cumulative, predictable, and distressing (eg, seasonal shortages and declining resources, declining wages, declining job opportunities, declining pasture lands, declining returns to labor, declining yields, and declining rainfall).

*Shocks* are natural, health-related, political, and economic events that people have no control over and result in income and nonincome losses (well-being)—impacts that are typically sudden, unpredictable, and traumatic such as pestilences, floods, and epidemics. Shocks can be idiosyncratic, affecting individual households (eg TB, human immunodeficiency virus [HIV]), or covariant, affecting communities or regions (eg, floods, pestilence, and coronavirus disease 2019 [COVID-19]).

*Human capital* refers to health, knowledge, and skills that a household possesses.

*Social capital* refers to relations that are based on exchange, trust, and reciprocity that households depend on.

*Physical capital* is assets such as buildings, machinery, and equipment. They are a measure of wealth.

*Natural capital* includes land and livestock.

*Financial capital* refers to cash, stock, and savings. It may take the form of savings, earnings (whether regular wages or one-off payments), access to loans, or money stored in saleable property such as livestock.

Households live in a “*vulnerability context*” that is characterized by shocks (COVID-19, job losses), trends (financial crises, climate change), and seasonality of jobs. The context is also shaped by transforming structures and processes such as laws, culture, and policies. *Transforming structures and policies* influence how people access and utilize the 5 capital assets, making the vulnerability context worse or better [24]. A policy on free TB diagnosis promotes early diagnosis and reduces frailty and loss of income due to TB disease.

*Livelihood* is dynamic and changes over time [2]. Households experience shocks continuously and employ various livelihood strategies and activities to maintain their well-being. The strategies and activities are influenced by social relations, human capital (ability to labor and skills), physical capital, and financial capital. Thus, having 5 capital assets does not necessarily translate into their effective use: Human and social capitals are required to fully utilize other capitals [24]. Households may exchange, sell, or utilize capital assets to generate income and ensure livelihoods through interacting with other households. They may adopt coping strategies to survive shocks or accumulative strategies to make savings that act as buffers against future shocks [17, 21, 23]. Coping strategies may be reversible or nonreversible and may determine livelihood outcomes: resilience or vulnerability to shocks.

## IMPACT OF TB ON THE 5 CAPITAL ASSETS

Each of the 5 livelihood capital assets has a direct, but distinct, association with TB. *Human capital* refers to health or capacity to work, knowledge, and skills in a household [22]. It is gained through formal or informal training. Formal education is expensive but provides better job opportunities and livelihood prospects [24]. TB affects human capital through reducing income and productive capacity, access to formal education resulting from financial constraints, and cessation of informal training following deaths of skilled people. Most people affected by TB are economically productive and contribute to household income prior to diagnosis. Large productivity and income losses occur when low-skill workers are affected by TB, as their income depends on their physical strength [22]. People who do not have medical insurance may cope by delaying seeking healthcare while some may first seek healthcare from private clinics and pharmacies [25]. When they are finally diagnosed, they may have exhausted financial resources and may be fragile [26, 27]. People lose productivity and income due to sickness, time spent in clinics, and work absenteeism by sick people and household members who accompany sick people to hospitals [2]. Household members either reduce working hours or stop working to assume noneconomic roles (eg, caring for sick people, housekeeping, and child rearing) that previously were done by the sick person. Hence, loss of household income coincides with periods when expenditure increases to cater for food and medical costs [8]. Productivity losses may be exacerbated when TB-related deaths occur and when TB occurs during productive cycles in settings where income is seasonal [2]. Policies that ensure early health seeking for TB and livelihood diversification may reduce socioeconomic impacts of TB while social educational assistance may improve future prospects of children in TB-affected households.

*Social capital* refers to relations that are based on exchange, trust, and reciprocity [13]. Social capital (support from close and extended family members, friends, and neighbors) is crucial during TB treatment. When TB affects human capital, households may draw on their social networks to help with child care, household chores, and food. Households make use of social capital for financial support, interest-free loans, and assistance on transport to get to clinics. Social capital therefore enhances resilience to TB. However, TB-affected households may not be able to reciprocate help and may fail to repay loans, reducing their creditworthy status [28]. This lowers their social capital. Social networks may also be disrupted by TB-related stigma, mental health, death of family members on which the networks are built, or abandonment by a spouse/family [24].

*Physical capital* refers to assets (tools, machinery). They are a measure of wealth and sale of physical assets may be a proxy for financial catastrophes. TB affects access to and maintenance of physical capital by diverting money toward treatment and care. However, TB-related deaths may result in early acquisition of physical and natural capital by children, preventing their effective utilization due to limited skills and poor maintenance [24].

*Natural capital* (land) is usually governed by the land tenure system. It may be sold, leased, or left fallow as a result of TB because households are unable to farm successfully due to reduced labor or skills. Unlike physical capital, land may be difficult to dispose since it may belong to the state. Urban land or plots usually have title deeds and can be rented out, sold, or used as collateral to get loans, whereas options are more limited in rural areas.

*Financial capital* refers to cash and savings. Households incur direct nonmedical (food and transport), direct medical (consultations, cost of ancillary medicines), and indirect costs (income loss). These costs are mostly out-of-pocket payments and they diminish financial capital.

Besides individual characteristics of each asset, there are trade-offs between them. Weighing these trade-offs is an ongoing process and influences coping strategies. Initially, households may borrow from friends, liquidate savings, or reduce number of meals (reversible coping strategies). Prolonged or sudden shocks that are greater than the capacity of households to cope may force financially stressed households to adopt nonreversible coping strategies (eg, borrowing at exploitative interest rates, selling productive assets, or withdrawing children from schools). Assets (physical and natural) may be sold to replenish financial capital [20, 29]. As households fail to recover from shocks, so does their capacity to replace assets. The dwindling asset base increases vulnerability to future shocks. Measures of impact of TB must attempt to measure this complex interplay. This may focus interventions aimed at preventing the downward spiral of households into poverty.

## APPLYING THE SLF TO MEASURE IMPACT OF TB

We propose to use the SLF and the indicators for each of the 5 capital assets and coping strategies as presented in Table 1. These were informed by a study investigating livelihoods in HIV-affected households using a participatory approach [29] and by qualitative interviews conducted with people on TB treatment in Zimbabwe [31]. Qualitative data gave insight into causal relationships among indicators, context-specific indicators with the greatest impact on livelihood, and reversible and nonreversible coping strategies and their impact on livelihood.

**Table 1.** Proposed Variables for Inclusion in the Model for Measuring Livelihood

Capital Assets	Variables That Increase Impact of TB	Variables That Increase Resilience to TB	Proposed Policies to Mitigate Impact of TB
Financial capital	Sale of assets^a^ Use of savings^a^ Failure to repay loans Pledging future crops/cattle/livestock^a^ Borrowing at exorbitant interest rates^a^ Reduction in household income	Social protection (cash, food, or both) Microfinancing^b^ targeting the person with TB, especially once TB treatment is completed	Medical insurance that covers chronic conditions and their sequalae Social protection schemes (eg, cash transfers) Improving financial inclusion and access to formal loan facilities (microfinancing schemes) Policies and programs aimed at diversifying livelihood strategies^c^ to mitigate impact of shocks
Physical capital	Sale of productive assets^a^ Failure to replace productive assets Replacing sold assets with inferior assets		
Natural capital	Reduction in land that is farmed (idle land/leasing) Sale of land or property Renting out of property (relocation of the household) Sale of cattle/livestock		
Social capital	Deterioration of relationships with neighbors or family members (stigma, loss of creditworthiness) Abandonment by spouse and/or family	Support from friends, neighbors, and family Psychosocial support Community education campaigns to combat TB-related stigma	Community awareness campaigns Provision of psychosocial care
Human capital	Death in a household Physical debility resulting in loss of labor for household Time spent caring for the TB affected household member Death of a household member due to TB Increased dependency ratio^d^ Education of children affected by TB in household^e^ Food insecurity (reduced number of meals/meat or fish in diet)	Food supplementation Free education Community-based TB treatment of TB	Medical insurance that covers chronic conditions and their sequalae Disability allowances/food vouchers to TB-affected households Workplace policies that allow paid sick leave Educational assistance to ensure children are in school Policies aimed at free, easily accessible, and earlier TB diagnosis and treatment

Abbreviation: TB, tuberculosis.

Coping strategies.

Microfinancing may be introduced to wean people off social protection and to build resilience of households. Microfinancing enables households to replenish physical and natural capital (replacing assets, acquiring new ones) and financial capital (amass income or savings). As a result of these accumulative strategies, households build buffers against future shocks.

Households explore other ways to raise income to mitigate impact of environmental shocks [30]. For example, households that earn their livelihood through farming crops may diversify into poultry or other crops or temporary migration to cities in search of work or engaging in microbusinesses.

Total number of people who contribute to household income or food security divided by the total number of dependents living in the household.

A derived variable obtained from moving children to cheaper schools and/or withdrawing children from school.

We propose a sampling strategy that identifies people affected by TB consecutively from TB registers. Household questionnaires will be administered either to the person affected by TB (if they are head of household) or the head of household. The household questionnaire captures questions on whether coping strategies (eg, selling assets, failure to repay loans, or spending savings) occurred following TB diagnosis (Table 1). In the presence of coping strategies, a score of 1 is assigned and in the absence of coping strategies, a score of 0 is assigned. Higher scores indicate loss of livelihood. Additional variables are derived during analysis. For example, children's education is affected by TB when they are transferred to cheaper schools or are withdrawn from school.

Given the dynamic nature of livelihood and long-term impacts of TB, including post-TB sequelae [32], repeat measurements are ideal. Coping strategies may evolve from short-term (spending savings) to long-term strategies (selling assets). The latter may cover for the former, hence cross-sectional surveys may not capture both coping strategies. Ideally, households should be interviewed at baseline and follow-up. The follow-ups could, depending on stage of treatment of person with TB at baseline interview, be conducted during either the continuation phase or post–TB treatment. This allows to investigate changes in livelihood during treatment and post–TB treatment, including reduced job opportunities due to post-TB disabilities (eg, chronic lung disease) [12, 32]. Post-TB sequelae reduce quality of life and job opportunities and result in healthcare-related costs even if TB is cured. Importantly because the unit of analysis is the household and the questionnaire is administered to the head of household, the impact of TB-related mortality is also captured.

The SLF was applied to develop indicators for HIV/AIDS, but the indicators were not used to calculate a composite measure [29]. However, it has frequently been used in research on agroforestry and climate change [20, 33, 34]. These studies have aggregated indicators from the 5 capital assets into composite measures using principal component analysis. Such composite variables were presented as spider diagrams. We propose to further aggregate the 5 capital assets variables into a dichotomous variable indicating loss of livelihood or resilience to facilitate measuring associations between loss of livelihood and explanatory variables. Importantly the proposed measure is not benchmarked against income and includes monetary and nonmonetary dimensions of well-being and coping costs. It is therefore holistic and multidimensional. While data on income are collected, it is not absolute income that is captured, but rather any changes in household income.

## BENEFITS OF THE PROPOSED MEASURE

The proposed measure can be used to determine loss of livelihood at any time during the TB episode. If used at onset of treatment, the measure may aid prioritization of most deprived people/households aiming for vertical equity whereby worse-off people/households are given more resources (social protection) so that they attain better health outcomes. At treatment completion, the measure may aid in identifying households that may be prioritized for TB sensitive social protection [35], because they experienced loss of livelihood and are at risk of secondary TB.

Social protection is an efficient, redistributive way to mitigate socioeconomic impact of chronic diseases (eg, TB), and is a key component of the End TB Strategy [36]. The most commonly used form of social protection is cash and/or food vouchers. One-dimensional measures (eg, TB treatment outcomes) are often used to measure the effect of social protection [37]. This approach may be too narrowly focused as it ignores the benefits of social protection beyond individual levels. Contributions by household members in terms of their productive time (caring for sick persons), money, and coping costs in ensuring successful outcomes are ignored [11]. While social protection may improve health outcomes, it acts at the household level to build resilience and reduce sale of assets [38]. Hence, a holistic household-level measure such as the one proposed may be ideal to evaluate social protection programs by focusing on protective effect of social protection against either loss of livelihood or dissavings (selling assets and borrowing).

The proposed measure adds a new dimension to capture monetary and nonmonetary losses of socioeconomic status due to TB. Hence, the proposed measure is likely to capture severity and complexity of damage caused by TB. This may inform targeted multisectoral interventions.

We acknowledge some limitations. First, we cannot rule out subjectivity in selecting indicators. However, the qualitative interviews we conducted helped to prioritize the indicators of livelihood and to establish causal relationships and sequencing of coping strategies in our setting. Second, all indicators are weighted equally to ease data analysis. Equal weighting may not reflect real-life situations and potentially underestimates the size of the estimate. However, given the interplay of capital assets, we assume our measure will not be affected greatly. Third, livelihood does not exist in a vacuum: it is influenced by the vulnerability context, processes, and structures, which are setting specific. This limits comparability across countries. Hence, context-specific indicators need to be developed and validated [21]. Often shocks affect households concurrently or sequentially and they synergize to influence negative livelihood outcomes [5]. Hence, socioeconomic impacts may be attributed not just to TB but to other shocks, which may be covariate (affecting regions) or idiosyncratic (affecting particular households) or both.

We have provided an alternative, holistic, and multidimensional measure of the household socioeconomic impact of TB. This multidimensional measure heightens attention to targeted multisectoral interventions in TB programs.

## Notes

***Author contributions*.** Conceptualized the study: C. T. and K. K. Critically reviewed the first draft and final draft, and read and approved the final manuscript: All authors.

***Financial support***. C. T. was funded by the Fogarty International Centre of the NIH (award number D43 TW009539). R. A. F. was funded by the Wellcome Trust (fellowship grant number 206316_Z_17_Z). R. M. G. J. H. was funded by a European Research Council fellowship.
